# Beyond the Monomer: Camphorquinone, TPO, and BAPO as Leachates of Light-Cured and 3D-Printed Dental Polymers—Radical Chemistry, Redox Signalling, and the Dose-Realism Gap

**DOI:** 10.3390/ijms27198583

**Published:** 2026-09-25

**Authors:** Marcin Mikulewicz, Edward Kijak

**Affiliations:** 1 Department of Dentofacial Orthopaedics and Orthodontics, Division of Facial Abnormalities, Wroclaw Medical University, Krakowska 26, 50-425 Wroclaw, Poland; 2 Department of Prosthodontics, Wroclaw Medical University, Krakowska 26, 50-425 Wroclaw, Poland; edward.kijak@umw.edu.pl

**Keywords:** photoinitiator, camphorquinone, acylphosphine oxide, 3D-printed orthodontic appliances, leachables, risk assessment

## Abstract

Research on the biological cost of resin-based dental materials has focused almost exclusively on residual methacrylate monomers. The photoinitiators—camphorquinone (CQ) with its amine co-initiator, and the acylphosphine oxides TPO and BAPO—remain partly unconsumed, can elute, and are seldom measured. Vat photopolymerisation, where acylphosphine oxides dominate, makes this omission material. We critically appraise the photochemistry, elution behaviour, molecular stress responses, and exposure data for CQ, TPO, and BAPO, weighted toward orthodontic appliances, and ask whether a margin of safety can be estimated. Within studies, TPO is 15- to 250-fold more cytotoxic than CQ, and neither TPO nor BAPO raised intracellular reactive oxygen species measurably in the two studies that measured it, so the CQ redox narrative cannot be assumed to transfer. Extractable TPO content of printed splint and orthodontic resins spans about a thousand-fold; the highest values exceed the TPO EC_50_ numerically, but exhaustive extraction is not an intraoral dose. No retrieved study has measured any photoinitiator in human saliva. Photoinitiator hazard is established in vitro; a clinically relevant, physiologically anchored margin of safety cannot currently be estimated reliably. TPO’s EU cosmetics prohibition alongside its continued intraoral use sharpens this asymmetry.

## 1. Introduction

Resin-based materials are the substrate of contemporary restorative dentistry and, increasingly, of orthodontics. Adhesives, bracket cements, bonded retainers, occlusal splints, and—since the arrival of direct printing—aligners themselves are methacrylate networks formed by light-driven free-radical polymerisation. Two generations of research have interrogated what escapes from those networks, and that research has overwhelmingly focused on monomers. Bisphenol A glycidyl dimethacrylate (BisGMA), urethane dimethacrylate (UDMA), triethylene glycol dimethacrylate (TEGDMA) and 2-hydroxyethyl methacrylate (HEMA) have been quantified in eluates, in artificial saliva, and in patient saliva [1]. The photoinitiator that converted the monomer into a network has almost never been given the same scrutiny.

This is not a small omission. Photoinitiators are, by design, unstable under light. Camphorquinone (CQ) is an α-diketone; the acylphosphine oxides diphenyl(2,4,6-trimethylbenzoyl)phosphine oxide (TPO) and phenylbis(2,4,6-trimethylbenzoyl)phosphine oxide (BAPO) undergo Norrish type I α-cleavage into acyl and phosphinoyl radicals [2]. They are added at fractions of a percent to a few percent by weight and are never fully consumed [3]; what remains is not covalently bound to the network. Every mechanistic reason to expect a leachable monomer is also reason to expect a leachable photoinitiator.

Three developments now make the question urgent rather than merely interesting in principle. First, the fastest-growing class of dental polymers—vat-photopolymerised printing resins—is initiated by acylphosphine oxides rather than CQ, and alternative initiators are increasingly used in restorative composites as well. The α-diketone chromophore that makes CQ work also makes restorations yellow, and its Type II bimolecular mechanism is radical-poor; acylphosphine oxides cure faster, more completely, and near-colourlessly [4]. The molecule whose biology is best characterised is therefore not the molecule to which a patient wearing a printed appliance is exposed. Second, direct-printed orthodontic appliances present an exposure geometry unlike any restorative: large surface area, 20–22 h of intraoral wear per day, weekly replacement, and treatment durations of one to two years. Third, and most sharply, TPO’s harmonised classification as a category 1B reproductive toxicant [5] triggered its outright prohibition in European cosmetic products from 1 September 2025, while it remains permissible in intraoral medical devices under a different regulatory logic [6]. The same molecule is now banned in a nail gel and allowed in an appliance worn against the oral mucosa for a year—a contrast in regulatory logic which is, by itself, evidence of neither safety nor harm.

The working assumption behind much of the literature—that photoinitiator leachates are “molecular stressors of oral tissues”—is a hazard claim dressed as a risk claim. This review separates the two. We ask what the radical chemistry actually predicts, what molecular stress responses are attributable to photoinitiators rather than borrowed from the monomer literature, what has genuinely been measured on the exposure side, and whether those two halves can be placed in the same equation. The scope is weighted toward orthodontics, where the exposure scenario is most extreme and the data thinnest. Our conclusion is deliberately uncomfortable: for the substances covered in this review, the hazard side is well described, the exposure side is close to empty, and a clinically relevant, physiologically anchored margin of safety cannot currently be estimated reliably.

This is a critical review rather than a systematic one, and the distinction is substantive. We have not pooled effect estimates, because the underlying studies are not commensurable: they differ in test article, extraction vehicle, exposure duration, and cell type to a degree that makes quantitative synthesis misleading rather than merely imprecise [7]. Instead, each cited study is appraised for what it actually demonstrates and, equally, for the substance it actually tested. We apply one rule throughout, and apply it strictly: a finding obtained with a monomer is reported as a finding about that monomer, never as a property of the photoinitiator system that accompanied it. Applying that rule turns out to remove most of the mechanistic scaffolding the field has built.

### How the Literature Was Searched and Appraised

Because several of the conclusions below are statements about what has not been measured, the search on which they rest is reported here so that their scope can be judged. PubMed/MEDLINE, Scopus, and the Web of Science Core Collection were searched from database inception to the final search date of 5 September 2026, without language restriction, combining photoinitiator terms (camphorquinone; TPO or diphenyl(2,4,6-trimethylbenzoyl)phosphine oxide; BAPO or phenylbis(2,4,6-trimethylbenzoyl)phosphine oxide; acylphosphine oxide; photoinitiator) with terms for release and biological effect (elution, leaching, extractable, residual, saliva, cytotoxicity, genotoxicity, reactive oxygen species, oxidative stress) and for the materials of interest (dental resin, composite, adhesive, 3D-printed or additively manufactured, aligner, splint, retainer). Reference lists of all included papers and of the systematic and scoping reviews identified were hand-searched (backward citation chaining). Included were peer-reviewed primary studies, systematic and scoping reviews, regulatory documents, and standards in which CQ, TPO, or BAPO was the test article or analyte, or in which the photoinitiator system of a dental polymer was identified; conference abstracts were excluded. Multiple papers from one research group were retained as separate studies and are identified as such where they are counted. Statements of the form “No study has measured” therefore refer to this search, these criteria, and this date: they describe the retrieved literature and are not proofs of absence, and they are phrased accordingly throughout.

## 2. What Is Actually in the Material, and Why

### 2.1. Camphorquinone and the Type II Mechanism

CQ absorbs between roughly 400 and 500 nm and, on excitation to its n,π* triplet, abstracts hydrogen from a tertiary amine co-initiator—typically 2-(dimethylamino)ethyl methacrylate (DMAEMA), ethyl 4-dimethylaminobenzoate (EDMAB), or N,N-dimethyl-p-toluidine—through an exciplex, generating the aminoalkyl radical that initiates propagation (Figure 1a). The mechanism is bimolecular and therefore inefficient: electron paramagnetic resonance shows that a CQ/amine composite produces substantially fewer propagating radicals per unit radiant exposure than a TPO composite, which is why a TPO formulation can cure twenty times faster and release at least four times less monomer under spectrally matched irradiation [8].

Inefficiency has a direct consequence for leachables. Mapping CQ consumption spectrophotometrically across systematically varied CQ/amine ratios and total initiator loadings shows that CQ consumption becomes less efficient as total initiator rises and as the amine/CQ ratio falls, and that above about 1.5 wt% overall initiator concentration, conversion and mechanical properties become independent of both concentration and ratio, while high CQ impairs light transmission [3]. The excess simply remains. Consistent with this, eluted CQ and its amine co-initiator 4-dimethylaminobenzoic acid ethyl ester correlate inversely with degree of conversion across nano-hybrid composites (r = −0.96, −0.88, and −0.69 for CQ in three composites; r = −0.87 for the amine) [9]. The unreacted photoinitiator reservoir is not an accident of manufacturing; it is a predictable function of formulation and cure.

Two ancillary points follow. The amine co-initiator is itself a leachable, and it is not innocuous: EDMAB gave an EC_50_ of 0.68 mM in human pulp-derived cells, of the same order as CQ itself, so a CQ system releases two bioactive species rather than one [10]. And the co-initiator’s fate is chemically distinct from CQ’s—the amine is consumed by hydrogen abstraction and converted to an aminoalkyl radical that is incorporated, whereas residual CQ both yellows the restoration and remains photochemically active in situ [4]. A restoration containing unconsumed CQ is, in effect, a small photosensitiser depot that continues to respond to intraoral light.

### 2.2. TPO and BAPO: Type I α-Cleavage

Acylphosphine oxides work differently. Ultrafast spectroscopy of TPO and BAPO shows excitation followed by rapid intersystem crossing into a hot triplet manifold from which α-cleavage generates phosphinoyl and benzoyl radicals on a timescale comparable to intersystem crossing itself; TPO crosses faster than BAPO, whose dynamics are more complex, and TPO photoproducts remain detectable on microsecond timescales [2]. The process is unimolecular and needs no co-initiator, and BAPO cleaves twice. In experimental Bis-GMA/TEGDMA composites, TPO at 0.27–1 wt% gave higher Vickers hardness (43.18 ± 1.7 HV at 1 wt%), higher diametral tensile strength (29.73 ± 4.8 MPa at 0.75 wt%), and, at 0.75 wt%, less yellowing than a CQ/DMAEMA system [11]. BAPO at 1 wt% gave the highest hardness of the systems compared in a parallel study (43.73 ± 3.52 HV), although CQ composites retained the highest flexural strength (77.3 ± 8.84 MPa), so the substitution is not uniformly advantageous [12]. Table 1 summarises the comparison. A narrative synthesis of the field reaches the same guarded conclusion: no single alternative matches CQ across all properties, and the drive for alternatives is explicitly motivated by CQ’s yellowing and biocompatibility [4].

The blue shift of acylphosphine oxide absorption relative to CQ has an under-appreciated safety consequence. A systematic review of 18 in vitro studies, nine of which used TPO, found that polywave light-emitting diodes improved degree of conversion in nine studies and microhardness in seven relative to monowave units [13], consistent with TPO under-activation by narrower monowave emission. Under-activation means unconsumed initiator, and unconsumed initiator is leachable initiator. The evidence was graded low quality, so this remains a mechanistically compelling rather than a firmly established link.

The two acylphosphine oxides should not be treated as interchangeable. BAPO carries two acyl groups and cleaves twice, giving a higher radical yield per absorbed photon; TPO undergoes faster intersystem crossing while BAPO shows more complex excited-state dynamics [2]. That difference has a toxicological corollary—in each of the three studies that tested both compounds side by side, BAPO, the compound generating more radicals, was the more cytotoxic of the pair [15,16,23]—but the link between radical yield in solution and potency in cells has not, to our knowledge, been tested directly. Formulation practice compounds this: acylphosphine oxides are often used with CQ synergistically rather than as replacements [4], so a patient may meet a Type I and a Type II initiator at once, with mixture effects already shown to be non-additive in both directions [19].

### 2.3. The Printed Orthodontic Appliance

Where the initiator system of a commercial vat-photopolymerisation dental resin has been identified analytically, it has been an acylphosphine oxide [21]. Formulation studies place TPO at 1–3 wt% in printable provisional resins; notably, raising TPO from 1 to 3 wt% left gingival fibroblast viability unchanged at 97.78–102.16% and produced no difference in degree of conversion at either post-cure time (*p* = 0.68 and *p* = 0.77), although 3 wt% gave the highest flexural strength and modulus and worse colour stability on ageing [14]. The only matched-loading comparison available places TPO, BAPO, and the liquid analogue TPO-L at 0.1 mol% in an identical DLP resin: L-929 fibroblast viability differed significantly across all three (*p* < 0.05), at 89.62 ± 4.93% for TPO-L, 84.45 ± 3.62% for TPO, and 74.16 ± 3.70% for BAPO [23]. Initiator substitution is therefore a realistic mitigation route, not a theoretical one. Thermoformed aligners, by contrast, are not photopolymerised at all and contain no photoinitiator—a comparison that anchors the whole discussion, since systematic review of thermoplastic aligners and retainers found no in vitro cytotoxicity or estrogenicity, while a single clinical trial reported elevated salivary bisphenol A after thermoplastic retainer wear [24].

The orthodontic exposure geometry deserves explicit statement, because it is what distinguishes this application from restorative dentistry. A restoration presents a small, largely occluded surface, partly protected by dentine—no cytotoxicity was detected for polymerised adhesives in a dentine barrier test using a 200 µm barrier [10]. A printed aligner presents a large, unshielded polymer surface in continuous contact with buccal, lingual, and palatal mucosa for 20–22 h a day; it is replaced weekly, so a fresh, maximally loaded surface is introduced repeatedly rather than once; and treatment runs for one to two years. Whatever margin the dentine barrier provides in operative dentistry is simply absent here. Several authors have raised cumulative exposure across an aligner series as a concern [22], and none of the studies we retrieved has quantified it; the one relevant experiment followed one aligner’s fifteen days of wear, not a thirty-aligner series.

## 3. The Reservoir: What Governs How Much Remains

If unreacted photoinitiator is the hazard’s source term, then the variables that govern it are the practical levers on exposure, and they sit almost entirely in post-processing rather than in printing.

Three quantities are distinguished throughout and should not be conflated. *Residual* photoinitiator is the mass of unconsumed parent compound present in the cured polymer, whatever its subsequent fate. *Extractable* photoinitiator is the fraction of that residue recovered into a defined vehicle under a defined regime—often an organic solvent, elevated temperature, or repeated exhaustive cycles—and is an upper-bound estimate of what could leave the material, not of what does. *Intraoral release* is the amount that actually enters saliva or reaches the mucosa during wear, and is the only one of the three that constitutes exposure; for CQ, TPO, and BAPO, it has not been measured in any study we retrieved. A fourth distinction cuts across all three: the analytical studies cited here quantified the parent compound against a TPO or BAPO reference standard [21,22], whereas the species generated on irradiation are phosphinoyl and benzoyl radicals and their recombination and oxidation products [2]. Cleavage products are therefore neither captured by parent-compound assays nor represented in the isolated-compound cytotoxicity data, which used the parent compounds as test articles [10,15]; the light-amplified cytotoxicity reported for the same initiators [16] is the only observation in this set that bears on them, and it does not identify the responsible species.

The most striking demonstration remains a study of 600 printed splint discs in which survival of RAW264.7 macrophages exposed to specimen extracts ranged from 9.1 ± 1.3% to 58.5 ± 5.9% across all combinations of two materials (Luxaprint OrthoPlus, DMG, Hamburg, Germany; V-Print Splint, VOCO, Cuxhaven, Germany), two wash systems, two post-polymerisation units, and three build orientations. Post-polymerisation unit (*p* < 0.001), wash system (*p* = 0.002), and material (*p* < 0.001) were all significant; build orientation was not (*p* = 0.406) [25]. The two extremes of that range differ in material as well as in wash system and post-polymerisation unit, so the six-fold span is the joint effect of material and finishing and cannot be attributed to finishing alone; the range within a single material is not reported. What the study does establish is that two post-processing variables under the user’s control were each independently significant, and that matching washing and post-cure to the material brought both materials to their lowest cytotoxicity [25]. That remains the single most actionable finding in this literature, even though the magnitude of the finishing effect on its own is not separable from the published data.

Component variables have since been separated. Post-cure duration titrated against cytotoxicity in printed aligners shows a plateau: 14 min of post-curing gave significantly lower day-7 gingival fibroblast viability than 20 or 40 min, but 40 min conferred no advantage over 20 [26]. Post-curing atmosphere matters less than commonly assumed: ambient-air and nitrogen post-curing of actual aligner geometry differed significantly only during the first minute of polymerisation, thereafter converging on near-complete double-bond conversion [27]. An oxygen-barrier approach using glycerol immersion produced the cleanest dose–response available, cutting residual methyl methacrylate release in printed denture base resin from 26.68 ppm at 0 min to 7.78 ppm at 30 min of UV post-cure (*p* < 0.001) [28]. Wash solvent interacts with resin identity: isopropyl alcohol favoured one resin and absolute ethanol another across 5, 10, and 30 min UV protocols, so there is no universal recipe [29]. Print orientation influenced sorption and solubility (*p* = 0.008) but not monomer elution (*p* = 0.774) [30].

Three cautions belong here. First, most of these studies measure conversion, residual monomer, or cytotoxicity rather than residual photoinitiator. One exception is decisive. In one printed orthodontic resin, HPLC quantification against a TPO reference standard gave extractable photoinitiator of 35.5 ± 3, 12 ± 1, 8 ± 0.5, 6 ± 0.5, and 5 ± 0.3 mg per kg of printed material after 1, 3, 6, 10, and 14 min of post-curing, respectively, while the amount released into a salivary simulant over 14 days fell from 25 ± 2.1 to 2.4 ± 0.2 mg/kg across the same range, in the same specimens whose cell viability rose from 62–69.5% at one minute of post-curing to above 80% at six minutes [22]. The claim that post-processing is the principal modifiable lever on photoinitiator exposure therefore rests on one directly measured dose–response, in one resin, obtained by a method for which no limits of detection or quantification are reported; for every other material, it remains an inference chained across studies. Second, degree of conversion is not a proxy for safety: printed resins with 83–93% conversion nonetheless produced significantly higher intracellular reactive oxygen species in human gingival fibroblasts than zirconia or CAD/CAM composite blocks, with the oxidative signal decoupled from residual monomer content [31]. Third, two separate findings in the same study of three orthodontic printing resins deserve to be kept apart: biocompatibility was acceptable except where the material was insufficiently post-cured, and, independently, fifteen days of ageing in a salivary simulant released residual resin and degradation products that increased cytotoxicity [22].

The implication is uncomfortable for a profession that has adopted printing rapidly. The variables that dominate leachable load—post-cure unit, wash system, solvent and temperature, oxygen barrier—are the least standardised in practice, are often substituted for cost or convenience, and are almost never revalidated by the clinic that changes them. Manufacturers validate a workflow; users routinely assemble a different one from components. On the evidence above, an unvalidated workflow substitution is not a minor deviation: post-cure unit and wash system were each independently significant determinants of extract cytotoxicity in the only factorial study available [25], and the profession has no mechanism for detecting when such a substitution has occurred.

## 4. Molecular Stress Responses—And to What They Are Actually Attributable

### 4.1. Camphorquinone: A Genuine Redox Mechanism

CQ has a real and reasonably well-characterised redox biology. Non-irradiated CQ produced growth inhibition and a reactive oxygen and nitrogen species burst over 0.5–2.5 mM, reaching 2342 ± 1108% of control after 90 min at 2.5 mM in L5178Y/TK^+^ mouse lymphoma cells, accompanied by DNA strand breaks and 8-oxo-2′-deoxyguanosine; critically, the resulting mutant frequency rise failed the global evaluation factor threshold for biological relevance under OECD Test Guideline 490 [17]. CQ-induced apoptosis in oral keratinocytes is accompanied by caspase-3/7 activation, phosphatidylserine externalisation, and DNA fragmentation, and is significantly reduced by exogenous glutathione, although visible-light irradiation and the reducing agent N,N-dimethyl-p-toluidine raised ROS without proportionally increasing apoptosis [32]. In dental pulp cells, CQ at 1 mM reduced viability to about 70% and at 2 mM to about 50%, activating ATM/Chk2/p53 and GADD45α, inducing haem oxygenase-1, p21, and cyclooxygenase-2, arresting cells in G2/M, and driving prostaglandin E2 through MEK/ERK, with N-acetylcysteine, catalase, and superoxide dismutase all attenuating the viability loss [33]. More recent work places CQ in a lipid-peroxidation and eicosanoid axis, regulating expression of cytosolic, secretory, and calcium-independent phospholipase A2 together with COX-2 at 2 and 3 mM [34], and shows that CQ alone at 1 mM up-regulates HMOX1 and down-regulates NOX4, while CASP3 required HEMA with CQ, BCL-2 fell with HEMA, and BAX was unchanged [35].

The mechanistic dissociation from monomers is explicit and important. In primary human pulp fibroblasts, TEGDMA depleted glutathione at 0.5–5 mM without raising ROS, whereas CQ raised ROS at ≥1 mM and affected glutathione only moderately, and only at the highest test concentration of 5 mM [18]. Monomer and photoinitiator injure cells by different routes. This single observation forbids the transfer of monomer redox data onto photoinitiators—a transfer that is nonetheless routine in the field.

Two further features of CQ toxicology deserve emphasis because they cut against alarm rather than for it. The genotoxic signal is oxidative and repairable rather than mutagenic: CQ produced strand breaks and 8-oxo-dG but failed the biological-relevance threshold in a regulatory mutagenicity assay [17], and glutathione co-treatment prevents both the DNA damage and the apoptosis [32]. Furthermore, the concentrations at which all of this occurs are millimolar. Every mechanistic CQ study cited above works in the 0.25–3 mM band, and none reports an effect below 0.5 mM. The shape of the dose–response anywhere near a plausible oral exposure is therefore entirely unknown, and no no-observed-adverse-effect level can be stated for CQ in oral cells.

### 4.2. TPO and BAPO: More Potent, with No Measurable ROS Increase in the Studies Available

Here, the received narrative does not survive inspection. On the potency side, two within-study comparisons are available and agree in direction: in human pulp-derived cells, EC_50_ values were 0.06 mM for TPO against 0.88 mM for CQ, 0.68 mM for EDMAB, and 0.50 mM for the CQ/EDMAB pair—TPO roughly fifteen-fold more potent than CQ on a molar basis [10]. In human oral keratinocytes and V79 fibroblasts, BAPO and TPO produced concentration-dependent cytotoxicity over the tested range of 1–50 µM, 50- to 250-fold more cytotoxic than CQ tested in the same study; in V79 Chinese hamster fibroblasts—the only line in which genotoxicity appeared—BAPO at 10 µM reduced proliferation to 19.8 ± 7.3% of control and induced 12 ± 1 micronuclei against 15 ± 1 for CQ at 2.5 mM and 6 ± 3 in controls [15]. Across seven commercial photoinitiators in four cell types, BAPO was the most cytotoxic and TPO-L and MBF the least, and 455 nm irradiation increased cytotoxicity to differing extents [16]; CQ was not among the seven, so that study ranks the acylphosphine oxides against each other, not against CQ. TPO was also the most genotoxic single substance in a comet-assay comparison in MRC-5 human foetal lung fibroblasts, ranking above BisGMA and CQ, and it amplified both antagonistic and synergistic effects in mixtures; notably, the rank orders diverged, with BisGMA rather than TPO the most cytotoxic in the same study [19].

However, in neither of the two studies that measured it did an acylphosphine oxide raise intracellular ROS. Popal and colleagues measured intracellular ROS/RNS with the DCFH assay in oral keratinocytes and V79 cells and found no increase over 1–50 µM, despite BAPO at 10 µM inducing redox-regulated enzyme messenger RNA comparable to CQ at 2.5 mM [15]; Van Landuyt and colleagues likewise detected no ROS from TPO while observing differential antioxidant-enzyme expression, and glutathione synthesis inhibition had only a minor effect [10]. Two studies, one probe chemistry, and three cell types constitute a consistent null under the conditions tested, not a demonstration that no oxidative mechanism exists: a ROS signal below the assay’s sensitivity, confined to a compartment the probe does not report, or arising only from photolysis products rather than the parent compound, would not have been detected. The compounds most relevant to printed appliances are therefore the compounds for which the reactive-oxygen mechanism is least supported by direct measurement. What the evidence supports for TPO and BAPO is thiol and electrophile reactivity inferred from enzyme-expression shifts, plus radical generation demonstrated spectroscopically in solution [2] and light-amplified cytotoxicity in cells [16]. The bridge between those two observations has not been built.

This matters more than a mechanistic footnote. If the acylphosphine oxides act by electrophilic or thiol-directed chemistry rather than by generating reactive oxygen species, then the antioxidant strategies that plausibly mitigate CQ toxicity—glutathione, N-acetylcysteine, ascorbate—should not be assumed to mitigate theirs, and the biomarkers used to detect injury need to change. It also means that the acylphosphine oxides may act at exposures that produce no oxidative-stress signal at all, so a study whose sole oxidative endpoint is ROS, reporting a null as one printed-aligner assessment did [36], has not demonstrated the absence of a photoinitiator effect. There is a further caveat worth stating rather than smoothing: the potency rankings are not all made on the same basis. Isolated-compound potency values [10] and comparisons across commercial photoinitiators [16] answer a different question from comparisons of finished resins at a fixed initiator loading. Molar and mass-based comparisons of compounds with very different efficiencies are not the same comparison, and the field has not been careful about which it is making. The 15-fold and 50- to 250-fold figures quoted above are each within-study ratios and are quoted only as such; a ratio formed by dividing an EC_50_ from one study by an effect concentration from another, obtained in a different cell type, exposure time, and assay, is not constructed anywhere in this review and should not be read out of Table 1.

### 4.3. A Borrowed Pathway Map

The canonical signalling chain invoked in this field—ROS to KEAP1–NRF2/ARE, to NF-κB, to mitogen-activated protein kinases, to mitochondrial depolarisation and apoptosis—rests on thiol-based sensing that is mechanistically plausible for an α-diketone and for electrophilic cleavage fragments alike [37], and recent reframing of NRF2–KEAP1 as a signal-resolution circuit rather than a simple antioxidant switch is directly relevant, since it distinguishes adaptive transient activation from injury [38]. But almost every step of that chain has been demonstrated with monomers, not photoinitiators. NRF2 activation and the antioxidant enzyme battery come from HEMA [39]; the NF-κB dataset—inhibition of LPS-driven p65 translocation with suppressed TNF-α, IL-6, and IL-10—is HEMA at 0–8 mM in macrophages [40]; double-strand-break evidence in oral cells comes from whole composite eluates, in which *γ*H2AX foci rose significantly for two of six composites and GC/MS identified twelve substances, five of them methacrylates, with TEGDMA reaching 1080 µM [41].

Table 2 maps this systematically. Of the mechanistic steps commonly recited, ROS generation and glutathione involvement have photoinitiator-direct evidence for CQ only; NRF2 nuclear translocation, NF-κB activation, p38 and JNK signalling, mitochondrial membrane potential collapse, and *γ*H2AX have no photoinitiator-direct evidence at all. Only ERK has been shown for CQ [33]. Presenting the full chain as photoinitiator biology is misattribution, and reviewers of this literature should treat it as such.

Two mechanistic domains are not merely under-evidenced but empty. Endoplasmic reticulum stress and the unfolded protein response are invoked occasionally and have not, in the literature we retrieved, been examined for any dental resin constituent, photoinitiator, or monomer; any discussion of them belongs in a hypothesis section, not a results synthesis. The same applies to effects on the oral microbiome and on mucosal barrier integrity: we found no study that examined photoinitiator effects on microbial community composition, tight junction protein expression, or transepithelial resistance, despite an aligner presenting exactly the sustained mucosal interface where such effects would matter. Cell-type coverage is similarly narrow—oral keratinocytes, gingival fibroblasts, pulp cells, and macrophages are represented, while we found no study exposing periodontal ligament cells or odontoblast-like cells to an isolated photoinitiator.

## 5. Dose Realism

### 5.1. What Has Actually Been Measured

Table 3 assembles the concentrations relevant to this question that we identified in the search described in the Introduction (subsection “How the Literature Was Searched and Appraised”) and could verify against the primary source, with matrix and provenance for each. Two studies have quantified extractable photoinitiator in printed intraoral appliance materials. Exhaustive extraction—up to eight 72 h cycles at 37 °C—with HPLC quantification across six printed splint and orthodontic resins yielded total extractable TPO of 44.0 ± 17.1, 80.6 ± 21.1, 127.4 ± 25.3, 2813.2 ± 348.0, 33,424.6 ± 8357.9, and 42,083.5 ± 3175.2 ng/mL: an approximately thousand-fold spread across commercially available products [21]. Expressed molar, that range is 0.13–121 µM. Numerically, the upper end exceeds the TPO EC_50_ of 60 µM in human pulp cells [10] and lies above 50 µM, the highest concentration at which TPO and BAPO have been tested in oral keratinocytes and V79 cells [15] (Figure 1b). That juxtaposition is made to display the size of the gap between what has been measured and what a risk estimate would require, not to compute a ratio: an exhaustive extract is a content per millilitre of extractant, not a concentration at the mucosa, and an EC_50_ is a 24 h response of a cell monolayer to the neat compound. The authors nonetheless calculated a margin of safety above unity for all cured materials, and noted that uncured material may exceed toxicological limits. Two qualifications are essential and were not obscured by the authors: the extraction is exhaustive and not physiological, and the study is manufacturer-affiliated. The second study quantified photoinitiator in three printed orthodontic resins at a common 14 min post-cure by HPLC against TPO and BAPO reference standards: extractables into 50:50 ethanol/water over 24 h were 5 ± 0.3 mg/kg (BAPO, Graphy TC-85DAC), 5 ± 0.3 mg/kg (TPO, NextDent Ortho Flex), and 12 ± 1 mg/kg (TPO, Freeprint Ortho), and the amounts released into a phosphate-buffered saline simulant over 14 days were 1.9 ± 0.3, 2.4 ± 0.2, and 5.6 ± 0.1 mg/kg; expressed as the dose ingested from a 2 g appliance, these fell well below the oral toxicity benchmarks cited there, and no limits of detection or quantification are reported [22].

A parallel observation from restorative materials is equally instructive. In an eluate study of bioactive liners, CQ was quantified at approximately 0.05 mg/mL (≈300 µM) with a human dental pulp stem cell EC_50_ of approximately 0.068 mg/mL (≈410 µM)—the extracted concentration reaching roughly three-quarters of its own EC_50_. But no organic substances at all were detected in the water eluates; the CQ figure is a methanol number [20]. The apparent margin and the apparent alarm are both artefacts of solvent choice.

On the truly physiological side, we identified only two human salivary datasets for constituents of light-cured or printed dental polymers, and neither measures a photoinitiator. UDMA rose from below detection at baseline to 4.04, 5.49, and 7.27 µg/L at 24 h, 7 days, and 14 days, respectively, in fifteen patients wearing direct-printed aligners [42]—0.015 µM, four orders of magnitude below the in vitro concentrations at which the same monomer class is studied. In vivo salivary sampling after restorative placement shows a release spike within 30 min, near-complete clearance of most parent monomers by 24 h, and degradation products at concentrations *higher* than the parents persisting for weeks [1]. Neither study looked for CQ, TPO, or BAPO.

### 5.2. The Vehicle Problem

The gap between extraction numbers and salivary numbers is largely a vehicle artefact rather than a material difference. Elution studies routinely use 75% ethanol/water as an accelerator [30], and comparison of aqueous and organic extraction of the same materials shows organic solvents dominating recovery [20]. Even in artificial saliva at a defined surface-area-to-volume ratio of 1.2 cm^2^/mL, quantitative LC–MS/MS found substantially greater release from one product than another at every time point out to three months, and untargeted analysis identified at least 67 putative compounds in one eluate at 24 h and three months—including UDMA that the manufacturer’s safety data sheet did not disclose [43]. Composition declared is not composition present. Notably, when elution and cytotoxicity of the same printed composites were measured together, eluates produced neither cytotoxicity nor cell-cycle effects, and in ethanol post-processed specimens released less than non-post-processed ones [44].

### 5.3. The Standardised Test Is Part of the Problem

It is tempting to treat the standards as the fixed point against which this heterogeneity is measured. They are not: the framework itself embeds assumptions that make photoinitiator exposure hard to see.

ISO 10993-5 defines in vitro cytotoxicity testing for medical devices and supplies the criterion that dominates this literature: a reduction in cell viability of more than 30% relative to control is regarded as a cytotoxic effect [45]. That binary threshold does an enormous amount of work: it is applied to extracts of very different provenance, ignores sub-cytotoxic molecular stress, and converts a graded response into a pass or fail that propagates through systematic reviews as though it were a measurement. Several of the printed-aligner studies discussed above turn on whether a viability figure fell just above or just below 70% [46]. The matched-initiator comparison makes the point sharply: TPO-L, TPO, and BAPO resins differed significantly from one another, yet all three passed, at 89.62%, 84.45%, and 74.16% viability, respectively [23]. A binary criterion calls all three the same.

ISO 10993-12 governs sample preparation and extraction, including the surface-area-to-volume ratios and vehicles that determine what appears in an eluate at all [47]. Its provisions are where the artificiality enters: the choice of a polar and a non-polar vehicle is intended to bracket worst-case release, and it succeeds—which is precisely why an ethanol or methanol number is a bracket rather than an exposure [20,30]. The standard is not at fault; the routine reporting of its output as though it approximated intraoral conditions is. Compliance is also weaker than it appears: 68% of printed-appliance cytotoxicity studies claimed conformity with ISO 10993-5 or -12, yet 42% of those performing extraction reported no surface-area-to-volume ratio at all [7].

ISO 10993-17 supplies the toxicological risk assessment framework within which a constituent-specific limit would be derived, and it is the standard that a photoinitiator justification under the MDR would ultimately invoke [48]. It presupposes a quantified constituent. ISO 7405 provides the dentistry-specific preclinical evaluation methods, including the dentine barrier test, in which a 200 µm barrier abolished the cytotoxicity of photoinitiator-containing adhesives [10]—one of the most important pieces of dose realism in the field, and one that has no analogue for a mucosa-contacting orthodontic appliance [49].

The composite effect is a testing architecture optimised to detect gross material toxicity under worst-case extraction, not to quantify a low-abundance constituent under physiological conditions. Only the second can support a risk statement about photoinitiators, and it is not what the field has been doing.

### 5.4. Can a Margin of Safety Be Estimated?

Not reliably, and not in a form that is clinically relevant or physiologically anchored—and the reasons are specific rather than rhetorical. A number can of course be written down, and one manufacturer-affiliated study did so from exhaustive extractables [21]; what cannot currently be done is to anchor the exposure term in a measured or modelled intraoral dose.

First, the measured and the threatening are not the same substances. Every cytotoxicity threshold available for CQ, TPO, and BAPO is for the neat compound applied to cultured cells. Every intraoral measurement is of a monomer. Placing a micromolar TPO EC_50_ beside a microgram-per-litre salivary UDMA concentration is a category error, and a table that does so without saying so invites a reviewer to say it instead.

Second, the analytical floor is unreported. The best exposure study available does not state limit of detection or quantification for HPLC-UV at 4 µg/L [42], and at these concentrations, “below the cytotoxic threshold” and “below detection” are not distinguishable. This is the general problem of medical-device chemical characterisation: required sensitivity at an analytical evaluation threshold of 1.5 µg/day frequently exceeds analytical capability, while calibration against the Carcinogenic Potency Database indicates that over 95% of substances outside the cohort of concern pose no excess cancer risk at a threshold based on 120 µg/day [50].

Third, no exposure model exists. Converting a salivary concentration into a daily intake for comparison against a threshold of toxicological concern—1800, 540, and 90 µg per person per day, equivalent to 30, 9, and 1.5 µg/kg body weight per day, for Cramer structural classes I, II, and III [51]—requires flow rate, clearance, and swallowing fraction. Salivary physiology does not supply one dilution factor: film velocity varies several-fold between intraoral sites, and flow varies further with stimulation [52]. We found no published calculation of this kind for a photoinitiator.

**Table 3:** Effect thresholds and measured concentrations relevant to photoinitiator exposure from dental and orthodontic polymers. The table is a register of what has been measured, not a risk comparison: block A lists effect thresholds for isolated compounds on cultured cells, block B lists measured concentrations that differ in vehicle, extraction regime, and biological matrix, and block C lists risk-assessment reference values. Rows are commensurable in units only, and no ratio between blocks is implied. Molar conversions use M_r_ 348.4 (TPO), 166.2 (CQ), and 470.6 (UDMA).

Substance	Value Type	Value	Matrix/Vehicle	Model or Setting	Ref.
*A. Effect thresholds—isolated compound applied to cultured cells; not exposures*					
TPO	EC_50_	0.06 mM (60 µM)	Cell culture medium	Human pulp-derived cells, 24 h	[10]
CQ	EC_50_	0.88 mM	Cell culture medium	Human pulp-derived cells, 24 h	[10]
EDMAB	EC_50_	0.68 mM	Cell culture medium	Human pulp-derived cells, 24 h	[10]
CQ/EDMAB	EC_50_	0.50 mM	Cell culture medium	Human pulp-derived cells, 24 h	[10]
TPO and BAPO	Active cytotoxic range	1–50 µM; 50–250× more potent than CQ in the same study	Cell culture medium	Human oral keratinocytes (OKF6/TERT2); V79 fibroblasts	[15]
BAPO	Micronucleus induction	10 µM: 12 ± 1 micronuclei (control 6 ± 3)	Cell culture medium	V79 fibroblasts only	[15]
CQ	EC_50_	≈0.068 mg/mL (≈410 µM)	Cell culture medium	Human dental pulp stem cells, WST-1, 24 h; dental liner study	[20]
*B. Measured concentrations—extraction and* in vivo *data; not intraoral exposure estimates*					
TPO	Total extractable content	44.0 ± 17.1 to 42,083.5 ± 3175.2 ng/mL (0.13–121 µM)	Water/isopropanol, 37 °C, up to eight 72 h exhaustive cycles	Six printed splint and orthodontic resins, HPLC; manufacturer-affiliated study	[21]
CQ	Eluted content	≈0.05 mg/mL (≈300 µM) in methanol; not detected in water	Methanol vs. water, 72 h	Bioactive liner discs, GC/MS	[20]
CQ and DMABEE	Elution vs. conversion	r = −0.96, −0.88, −0.69 (CQ, three composites); −0.87 (DMABEE)	Ethanol/water, 7 d, 37 °C	Nano-hybrid composites, GC/MS	[9]
UDMA	Salivary concentration in vivo	not detected → 4.04 → 5.49 → 7.27 µg/L (0–14 d); 7.27 µg/L ≈ 0.015 µM	Stimulated human saliva	15 patients, direct-printed aligners, HPLC-UV	[42]
Methacrylates and degradation products	Salivary kinetics in vivo	Peak <30 min; degradation products exceed parent monomers and persist for weeks	Human saliva	Restorative placement, LC–MS	[1]
CQ, TPO, or BAPO	Concentration in human saliva	No published data identified	—	—	—
*C. Risk-assessment reference values*					
Any device constituent	Threshold of toxicological concern	1800/540/90 µg per person per day; 30/9/1.5 µg/kg bw/day (Cramer I/II/III)	Oral intake	Risk-assessment framework	[51]
Any device constituent	Analytical evaluation threshold	1.5 vs. 120 µg/day; >95% of non-cohort-of-concern substances below excess-risk at 120 µg/day	Chemical characterisation	Medical-device risk assessment	[50]

Fourth, the underlying studies are not commensurable. A scoping review of 25 cytotoxicity studies of printed orthodontic aligners and retainers found that 42% of those with an extraction step never reported surface-area-to-volume ratio, extraction duration ranged from 24 h to 14 days, and 76% relied on a single metabolic assay [7]. Without surface-area-to-volume, a reported eluate concentration cannot be converted into mass released per appliance, and therefore cannot become a dose at all.

### 5.5. The Orthodontic Exposure Scenario

Orthodontics is where the mismatch between exposure geometry and evidence is worst. Several directly printed aligner resins reduced human dental pulp stem cell viability more than thermoformed or polyamide-12 systems, with material-, dose-, and time-dependence and non-linear, hormesis-like responses in a subset of materials [46]; a review of retainer materials identifies photopolymer resins as the most cytotoxic class while finding no clinical endocrine signal [53]. Against this, the first biocompatibility assessment of a printed aligner found no cytotoxicity at 20%, 10%, or 5% *v*/*v* eluate, no estrogenicity, and—directly relevant here—no change in intracellular ROS-reducing capacity [36]. A critical review of the field puts the evidence base at eleven studies covering three marketed resins, two of them by a single investigation each, and calls explicitly for chemical characterisation of eluates [54]. Claims about “printed aligners” as a class are, at present, claims about one resin.

Background exposure compounds the uncertainty. Photoinitiators as a chemical class are ubiquitous environmental contaminants detected in human serum and breast milk [55], and market surveillance after the EU cosmetics prohibition found TPO still present in 90% of thirty gel nail polishes at 1.42–6.43% mass fraction, none of it declared on the label [56]. Dental exposure is one of several converging sources, and none of the dental risk assessments accounts for the others.

There is, finally, an instructive precedent for how the missing study should look. A prospective cohort of 211 children showed urinary bisphenol A rising 86% two days after placement of BisGMA-based restorations (95% CI 42–143%), with a non-significant trend toward larger increases where more than four surfaces were treated (112%, 95% CI 53–194%) than with four or fewer (50%, 95% CI −2–130%), and a return to baseline by four weeks [57]. That design—a defined exposure, a validated assay, repeated sampling, and a demonstrated washout—is what is required for TPO in aligner therapy, and what has not yet been published. Its result is also the right kind of answer for a hazard-versus-risk argument: a real, measurable, transient systemic exposure, neither dismissed nor inflated.

## 6. The Regulatory Asymmetry

TPO’s harmonised classification was revised to Repr. 1B (H360Fd, “may damage fertility; suspected of damaging the unborn child”) and Skin Sens. 1B (H317) by the 21st adaptation to technical progress of the CLP Regulation, applying from 1 September 2025 [5]. Under Article 15 of the Cosmetics Regulation, this classification triggers automatic prohibition, effected by the addition of TPO (CAS 75980-60-8; EC 278-355-8) to Annex II as entry 1731, applying from the same date [58]. The reversal is stark: the Scientific Committee on Consumer Safety had previously labelled TPO safe in nail modelling products at up to 5.0%, describing it as a moderate skin sensitiser [59]. Nothing new was measured; the same reproductive-toxicity dataset—testicular atrophy, reduced testis weight, and oligo- to azoospermia in rats, with 28-day and 90-day NOAELs of 50 and 100 mg/kg body weight per day, respectively, and negative genotoxicity in standard assays—supported a recommendation of only Repr. 2 in a contemporaneous non-EU assessment [60]. The classification changed; the toxicology did not.

Medical devices operate under a different logic. Regulation (EU) 2017/745 does not prohibit CMR category 1A or 1B substances. Above 0.1% weight by weight in invasive and body-fluid-contacting devices, it permits them where justified, requiring an estimate of patient exposure, an analysis of possible alternative substances, materials, or designs, and an argument as to why substitution would compromise performance or the benefit–risk ratio, together with labelling of the substance on the device or packaging [6]. Cosmetics regulation is hazard-based and categorical; device regulation is risk-based and conditional. Both are defensible on their own terms, and the difference between them is precisely the hazard–risk distinction this review applies: the cosmetics decision is a hazard classification acting through a categorical rule, and it says nothing about the intraoral risk of TPO, which only an exposure estimate could establish. Their coexistence is nonetheless uncomfortable when the two products deliver the same molecule to adjacent tissues for comparable durations, because the risk-based pathway is the one that requires exposure data, and those data have not been generated.

The practical difficulty is that the MDR pathway requires precisely the input this review has shown to be missing. A justification under §10.4 rests on an exposure estimate. For TPO in a printed orthodontic appliance, no measured intraoral exposure has been published, the extractable content varies a thousand-fold between products [21], and the substitution analysis has a plausible answer available—TPO-L or alternative initiator systems [23]—that has not been systematically evaluated for orthodontic use. Whether a given printed appliance crosses the 0.1% *w*/*w* threshold is a formulation question that manufacturers can answer and the literature cannot: reported TPO loadings of 1–3 wt% in printable resins [14] are an order of magnitude above it, though the relevant figure is content in the finished device, not the vat.

Two consequences follow for practitioners rather than regulators. Manufacturers hold information the published literature does not—the identity and weight fraction of the initiator system, and residual content after the validated workflow—and clinicians are entitled to ask for it, particularly given that safety data sheets have already been shown to be incomplete for at least one printed dental resin [43]. Moreover, where an equivalent thermoformed appliance exists and is clinically appropriate, it presents no photoinitiator at all; that is not an argument against direct printing, whose clinical advantages are real, but it is the correct comparator against which a printed appliance’s incremental exposure should be judged.

## 7. What Would Settle This

The agenda follows from the gaps. First, and by a wide margin most important: validated LC–MS/MS quantification of CQ, TPO, and BAPO in patient saliva during appliance wear, with limits of detection and quantification reported. Until one such study exists, every risk statement here is extrapolation. Second, residual photoinitiator quantified across a post-cure gradient in resins other than the single one for which this has been done [22], by methods whose limits of detection and quantification are reported, closing a chain that is currently evidenced for one resin only. Orthodontic printing materials have also been assayed at one post-cure (85% fibroblast survival after 30 min) without the paired initiator measurement [61]. Third, mechanistic work on TPO and BAPO that does not assume the CQ mechanism: thiol adduct formation, KEAP1 cysteine modification, and NRF2 reporter activity in oral cells. Fourth, exposure modelling that propagates real variability in salivary flow and clearance rather than a point estimate. Fifth, reporting discipline—surface-area-to-volume ratio, extraction vehicle and duration, and at least one non-metabolic assay—absent from much published work [7].

## 8. A Conceptual Model for Clinical Risk Assessment

The gaps listed above can be organised into a single chain that a clinical risk assessment for a photoinitiator would have to traverse, and stating the chain makes explicit which links can be populated now and which cannot. It follows the structure of ISO 10993-17 and ISO 10993-18 [48,62]: chemical characterisation yields a constituent-specific release, exposure assessment converts release into dose, and dose is compared with a health-based limit.

Link 1, the source term, is the residual parent photoinitiator per unit mass of the finished device after the validated post-processing workflow, quantified with stated limits of detection and quantification. It is measurable now and has been measured for one resin across a post-cure gradient [22]. Link 2 is release under physiological conditions: mass released per day into a saliva-like medium at the surface-area-to-volume ratio of the actual appliance and at 37 °C, reported as a kinetic profile rather than a single time point, and distinguishing the parent compound from its cleavage products. It is partly populated [22], but the surface-area-to-volume ratio needed to convert it into mass per appliance is often unreported [7]. Link 3 is the intraoral concentration, obtained by dividing release by the salivary volume that actually contacts the appliance, propagated over a salivary flow that varies several-fold between sites and with stimulation [52] and is negligible during sleep [63], when an aligner is worn but clearance is minimal; any such model must carry a distribution, not a point estimate. Link 4 is the measured salivary concentration: LC–MS/MS quantification of CQ, TPO, and BAPO in patient saliva during wear, with appliance series, sampling times, and limits of quantification reported, for which the bisphenol A cohort [57] is the design template. This has not been done for any photoinitiator. Link 5 is the systemic dose, the swallowed fraction of salivary concentration times flow plus transmucosal uptake, expressed in µg per kg body weight per day. Link 6 is the comparison of that dose with a limit: a tolerable intake derived from the reproductive-toxicity NOAELs [60] or, absent compound-specific data, the threshold of toxicological concern for the relevant Cramer class [51], with the analytical evaluation threshold [50] setting the sensitivity the assay must reach.

Only links 1 and 2 are populated today, each by one or two studies. A margin of safety requires link 6 in the numerator and link 5 in the denominator; the extraction-based margin reported for cured splint materials [21] substitutes link 2 for link 5 and is therefore a screening bound, not a clinical estimate. The chain also identifies where the mucosal-contact geometry of an aligner departs from the ingestion model that the threshold of toxicological concern assumes: for a device worn against tissue, local concentration at the mucosa (link 3) may matter more than systemic dose (link 5), and no local-effect benchmark for a photoinitiator exists in the studies retrieved. Stated this way, the list of gaps becomes an ordered programme, and link 4 is the experiment that populates links 3, 5, and 6 at once.

## 9. Conclusions

Photoinitiators are bioactive leachates, and treating them as inert adjuncts to the monomer question is no longer tenable. However, the evidence does not currently support labelling them molecular stressors of oral tissues in patients, and this review declines to do so.

Three conclusions are firm. Isolated-compound hazard is established and, in the two within-study comparisons available, is greater for the acylphosphine oxides than for camphorquinone, by roughly fifteen-fold for TPO on a molar EC_50_ basis and by 50- to 250-fold for TPO and BAPO in oral keratinocytes. The redox mechanism that dominates discussion of this field belongs to camphorquinone, not to the acylphosphine oxides that have replaced it in printing resins; TPO and BAPO are more potent, and in the two studies that measured intracellular ROS, neither produced a measurable increase; the downstream NRF2, NF-κB, and mitochondrial pathways commonly attributed to photoinitiators have been demonstrated only for monomers. Furthermore, exposure is essentially unmeasured: extractable TPO from printed appliances varies a thousand-fold between products, the highest values numerically exceeding in vitro effect thresholds under an exhaustive extraction that does not represent intraoral exposure, while in the literature we retrieved, no photoinitiator concentration has been reported from a human mouth.

The regulatory asymmetry created by TPO’s prohibition in cosmetics alongside its permitted intraoral use is therefore not a paradox to be explained away. It is a prompt. The device pathway that permits TPO requires an exposure estimate, and the profession cannot presently supply one. Post-processing remains the strongest available control—post-cure unit and wash system were each independently significant determinants of extract cytotoxicity alongside material choice [25], and residual TPO fell with post-cure time in the one resin in which it was measured [22]—and initiator substitution is a realistic alternative. Both are prudent now. Neither substitutes for measuring what patients are actually exposed to.

The honest formulation, and the one this review recommends the field adopt, is narrow: photoinitiator leachates are demonstrable hazards in vitro whose intraoral exposure has not been quantified in any published study we could identify, so statements of either safety or harm currently exceed the evidence. That is a less satisfying conclusion than either reassurance or alarm. It is also the only one the data support, and it identifies the study that would change it.

## Figures and Tables

**Figure 1:**
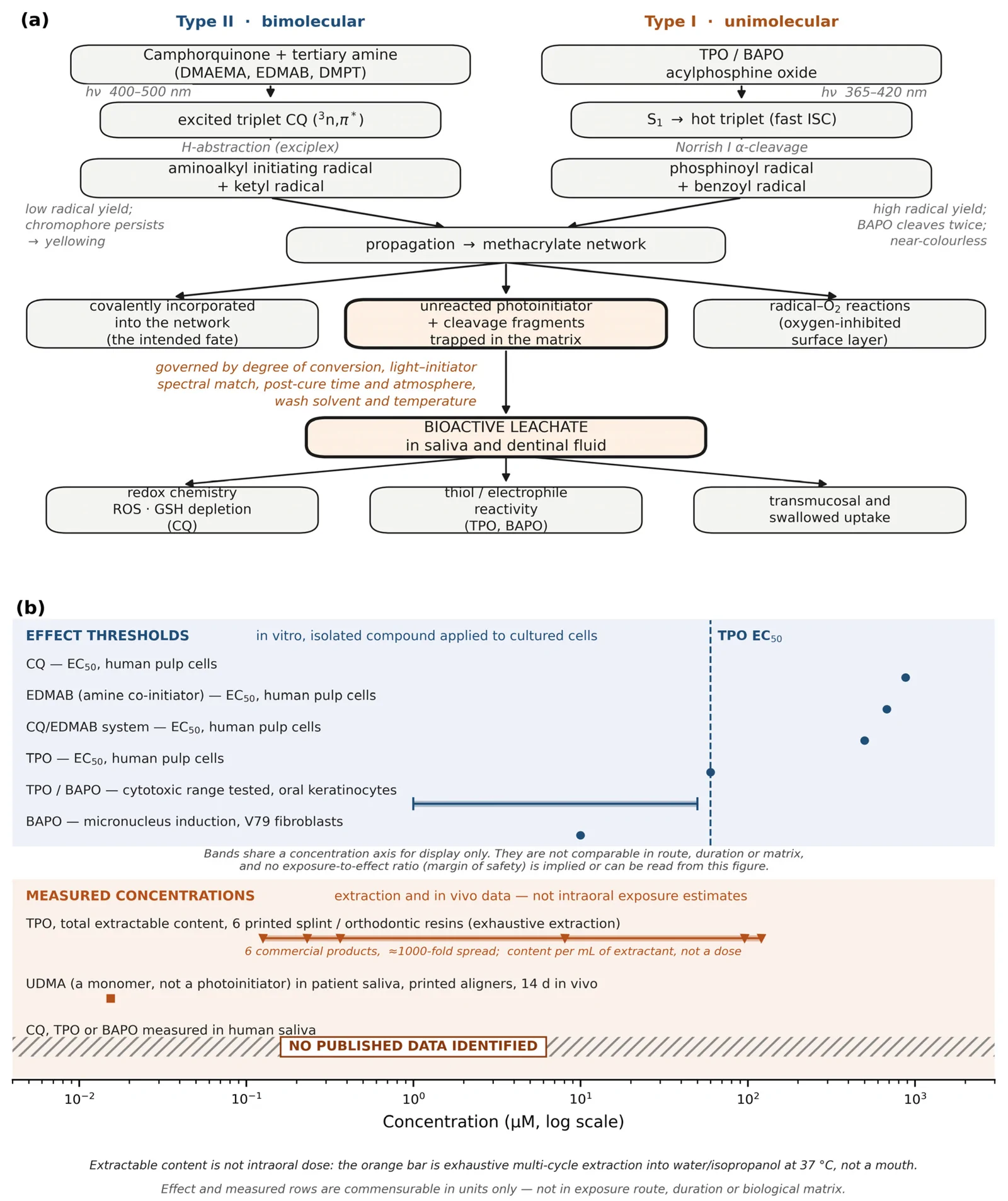
Photoinitiator chemistry and the dose-realism gap. (**a**) Type II camphorquinone/amine initiation proceeds by bimolecular hydrogen abstraction and is radical-poor, leaving an α-diketone chromophore that persists and yellows; Type I acylphosphine oxides (TPO, BAPO) initiate by unimolecular Norrish type I α-cleavage with high radical yield. In both systems, the fraction of initiator that is not consumed remains physically trapped rather than covalently bound, and becomes available for elution; the size of that reservoir is governed by degree of conversion, spectral matching, post-cure, and washing. (**b**) Effect thresholds for the isolated compounds (upper band) and the concentrations that have actually been measured (lower band), drawn on a shared logarithmic molar axis for display only; the bands are not comparable in exposure route, duration, or biological matrix, and no exposure-to-effect ratio is implied. Total extractable TPO content of six commercial printed splint and orthodontic resins spans about three orders of magnitude and numerically exceeds the TPO EC_50_ at its upper end, but the value is obtained by exhaustive multi-cycle extraction into water/isopropanol rather than from a mouth. In the literature retrieved, no photoinitiator concentration has been reported from human saliva. In (**b**), blue symbols denote effect thresholds, orange symbols denote measured concentrations, and the dashed vertical line marks the TPO EC_50_ (60 µM).

**Table 1:** Comparison of the three photoinitiators considered in this review. EC_50_ and active-range values are for the isolated compound applied to cultured cells and are not intraoral exposures; potency ratios are quoted only where both compounds were tested in the same study. “None reported” denotes an absence of published data, not a negative finding.

Property	Camphorquinone (CQ)	TPO	BAPO
Photoinitiator class	Type II α-diketone	Type I acylphosphine oxide	Type I bis(acyl)phosphine oxide
Initiation mechanism	Bimolecular H-abstraction from a tertiary amine via an exciplex [4]; fewer radicals per unit radiant exposure than TPO [8]	Unimolecular Norrish type I α-cleavage [2]	Unimolecular Norrish type I α-cleavage; two acyl groups, cleaves twice [2]
Co-initiator required	Yes (DMAEMA, EDMAB, DMPT)	No	No
Absorption relative to CQ	Visible, ≈400–500 nm [4]	Blue-shifted [4]; under-activated by monowave LEDs [13]	Blue-shifted; as TPO [13]
Typical loading in dental resins	0.5–3 wt% total initiator at CQ/amine 3:1 to 1:3 [3]	0.27–3 wt% [11,14]	0.25–1 wt% [12]
Principal driver of use/disuse	Displaced because of yellowing and low radical yield [4]	Faster, more complete cure; near-colourless [4,11]	Highest hardness of systems compared; not uniformly superior to CQ [12]
Cytotoxic potency, oral or pulp cells	EC_50_ 0.88 mM (human pulp-derived cells) [10]	EC_50_ 0.06 mM (human pulp-derived cells; within-study comparison with CQ) [10]; active 1–50 µM (oral keratinocytes; within-study comparison with CQ) [15]	Active 1–50 µM [15]; most cytotoxic of seven photoinitiators tested in one study (CQ not included), TPO-L and MBF least [16]
Reactive oxygen species in cells	Yes; 0.5–2.5 mM [17], ≥1 mM [18]	No increase detected under the conditions tested [10,15]	No increase detected under the conditions tested [15]
Genotoxicity signal	Comet and 8-oxo-dG positive, 0.5–2.5 mM; mutagenicity negative by OECD TG 490 [17]	Comet positive in MRC-5 human foetal lung fibroblasts; most genotoxic but second most cytotoxic of six substances [19]	Micronuclei at 10 µM in V79 cells only [15]
EU harmonised CMR classification	None	Repr. 1B (H360Fd); Skin Sens. 1B (H317), applying 1 September 2025 [5]	None
Extractable or released photoinitiator measured from a cured device	Detected only in organic solvent; not detected in water eluates [20]	44–42,083 ng/mL (0.13–121 µM) total extractable content across six printed splint and orthodontic resins [21]	5 ± 0.3 mg/kg extractable (50:50 ethanol/water, 24 h) and 1.9 ± 0.3 mg/kg released into PBS over 14 d, one printed orthodontic resin (Graphy TC-85DAC) [22]
Dental studies with a biological endpoint	Many	Few	Essentially none; published dental work is mechanical and optical only [12]

**Table 2:** Provenance of the mechanistic evidence commonly presented as photoinitiator biology. “Photoinitiator-direct” means that the cited study tested CQ, an amine co-initiator, TPO, or BAPO as the test article; “monomer-only” means the step has been demonstrated with HEMA, TEGDMA, BisGMA, UDMA, or a whole eluate. The right-hand column states what may legitimately be claimed. “None” and “not demonstrated” denote that no retrieved study tested the step with a photoinitiator as test article; they do not denote that the step was tested and found absent.

Mechanistic Step	Photoinitiator-Direct Evidence	Monomer-Only Evidence	What May Be Claimed
Reactive oxygen species generation	CQ, ≥1 mM [17,18,33]; no increase detected for TPO and BAPO under the conditions tested [10,15]	Whole printed-resin eluates raise ROS in gingival fibroblasts [31]	Established for CQ; no increase detected for TPO and BAPO in the two studies that measured it
Glutathione depletion/thiol reactivity	CQ apoptosis significantly reduced by glutathione [32]; glutathione synthesis inhibition had only a minor effect for TPO [10]	TEGDMA depletes GSH without raising ROS [18]	Established for CQ; thiol reactivity of TPO and BAPO inferred only
KEAP1–NRF2/ARE activation	None. Downstream ARE outputs (HO-1) induced by CQ [33,35]	HEMA activates NRF2 with a full antioxidant enzyme battery [39]	Not demonstrated for any photoinitiator
NF-κB signalling	None	HEMA blocks LPS-driven p65 translocation, 0–8 mM [40]	Not demonstrated for any photoinitiator
MAPK signalling	ERK only: U0126 blocks CQ-induced COX-2 and PGE_2_ [33]	—	ERK established for CQ; p38 and JNK untested for any photoinitiator
Mitochondrial depolarisation/intrinsic apoptosis	None	Not covered by the studies appraised here	Not demonstrated for any photoinitiator
DNA strand breaks/oxidative base lesions	CQ, comet and 8-oxo-dG, 0.5–2.5 mM [17]; TPO, comet in MRC-5 human foetal lung fibroblasts [19]	Composite eluates [41]	Established for CQ; single dataset for TPO
*γ*H2AX double-strand breaks	None	Composite eluates in gingival fibroblasts [41]	Not demonstrated for any photoinitiator
Micronucleus formation	BAPO at 10 µM in V79 cells only; not seen in human oral keratinocytes [15]	—	Established for BAPO in one non-oral line
Eicosanoid/inflammatory output	CQ induces COX-2 and regulates PLA_2_ isoforms at 2–3 mM [34]; PGE_2_ at 1–2 mM and 8-isoprostane at 1–3 mM [33]	—	Established for CQ
Endoplasmic reticulum stress/UPR	None	None	Not tested for any dental resin constituent in the studies retrieved
Oral microbiome/mucosal barrier	None	None	Not tested for any dental resin constituent in the studies retrieved

## Data Availability

No new data were created or analysed in this study. Data sharing is not applicable to this article.

## Abbreviations

ARE, antioxidant response element; BAPO, phenylbis(2,4,6-trimethylbenzoyl)phosphine oxide; BisGMA, bisphenol A glycidyl dimethacrylate; CMR, carcinogenic, mutagenic, or toxic for reproduction; CQ, camphorquinone; DMAEMA, 2-(dimethylamino)ethyl methacrylate; EDMAB, ethyl 4-dimethylaminobenzoate; HEMA, 2-hydroxyethyl methacrylate; MDR, Medical Device Regulation; ROS, reactive oxygen species; TEGDMA, triethylene glycol dimethacrylate; TPO, diphenyl(2,4,6-trimethylbenzoyl)phosphine oxide; TTC, threshold of toxicological concern; UDMA, urethane dimethacrylate.
